# Free testosterone value before radical prostatectomy is related to oncologic outcomes and post-operative erectile function

**DOI:** 10.1186/s12885-018-5148-1

**Published:** 2019-01-18

**Authors:** Tian Li, Xiangzhou Sun, Liheng Chen

**Affiliations:** 1 Department of Urology, the Fifth Affiliated Hospital of Guangzhou Medical University, 621 Gangwan RD, Huangpu district, Guangzhou, 510700 China; 2 Minimally Invasive Technique and Product Translational Center, Guangzhou Medical University, 621 Gangwan RD, Huangpu district, Guangzhou, 510700 China; 3 Department of Urology, the First Affiliated Hospital of Sun Yat-Sen University, 58 Zhongshan Second Rd, Yuexiu Dis, Guangzhou, 510080 China; 4 Department of Biomedical Engineering, Key Laboratory of Biomaterials of Guangdong Higher Education Institutes, Jinan University, 601 Huangpu Rd, Tianhe Dis, Guangzhou, 510632 China

**Keywords:** Biochemical recurrence (BCR), Erectile dysfunction (ED), Free testosterone (FT), Prostate cancer (PCa), Radical prostatectomy, Total testosterone (TT)

## Abstract

**Purpose:**

To investigate whether free testosterone (FT) prior to radical prostatectomy was related to post-operative oncologic outcomes, erectile function and continence.

**Methods:**

The data of 586 patients with available information underwent treatment in our center was retrospectively reviewed. Total testosterone (TT) was tested by chemiluminescence immunoassay, and FT value was calculated using Vermeulen’s formula. Post-operative continence and erectile function were evaluated by the requirement of pad and the IIEF-5 score at 12 months.

**Results:**

The median TT and FT value was 344 ng/dL (interquartile, IQR 314–374) and 6.9 ng/dL (IQR 6.4–7.3), and 106 patients (18.1%) and 152 patients (25.9%) were evaluated as having low TT and low FT based on current guidelines. Low TT and FT value were both related to older age (both *p* < 0.001), concomitant diabetes (*p* = 0.018 & 0.049), higher possibility of pre-operative erectile dysfunction (ED, both *p* < 0.001), higher pre-operative PSA value (both *p* < 0.001), higher clinical stage (both *p* < 0.001) and higher Gleason score in biopsy (both *p* < 0.001). Low FT was related to higher risk for pT3 (*p* = 0.020) and high Gleason score (*p* = 0.011) in logistic regression. The median follow-up duration was 52 moths (IQR 29–67) and FT was found to be an independent risk factor for biochemical recurrence (*p* = 0.005). In logistic regression TT was related to pre-operative ED (*p* = 0.010) and FT was related to post-operative ED (*p* = 0.001).

**Conclusion:**

Low FT value before radical prostatectomy was related to adverse pathological outcomes, biochemical recurrence and post-operative ED.

## Background

Androgen-deprivation treatment (ADH) is widely used in the treatment of prostate cancer (PCa) [1], and the longstanding concerns regarding testosterone and PCa has drove investigators to focus on the role in tumor development and the predictive value on prognosis [2, 3]. In decades there’s controversy in current studies focusing on the relationship between pre-operative testosterone value and the pathological and survival results in patients underwent radical prostatectomy (RP). Several literatures reported a poor prognosis in patients with high testosterone value, but more studies demonstrated the negative predictive role of low testosterone value [4–6]. Various mechanisms were raised, including the saturation model [7], the hypothesis of the development of hormonal-refractory tumors in androgen-depleted environment [8], and the corresponding metabolic disorders which may modulated PCa aggressiveness [9].

Free testosterone (FT) accounts for about 1–3% of total testosterone (TT). It’s generally regarded that decreased FT level was related to the presence of metabolic syndrome (MetS) [10, 11] and previous literatures proposed the regular test of FT in screening for hypogonadism [12]. Currently there’s been a few studies about FT in patients with PCa and the link between FT value and the aggressiveness of PCa has been demonstrated [13–16]. It’s notable that up to now no information about prognosis and quality of life after RP was available.

In the past several years, hormonal tests including TT and FT were regularly performed in patients before radical prostatectomy in our center. In this study, we collected the information of these patients to investigate whether there’s a relationship between FT and prognosis, erectile function and continence after surgery.

## Methods

### Patients’ enrollment and treatment

This was a retrospective study with approval by institutional review board of Fifth Affiliated Hospital of Guangzhou Medical University and the written consent from each participant. Patients underwent radical prostatectomy from Jan 2010 to Jan 2016 were analyzed. The exclusion criteria included incomplete data (8), a follow-up duration of less than 12 months (36), inability for questionnaires (in Chinese) (6), and patients’ refusal for participation (17). Ultimately 586 patients were included. There’s no difference between the patients included and the patients excluded in terms of TT and FT value, PSA value, tumor stage and Gleason score (data not shown).

All patients were diagnosed after trans-rectal ultrasound guided biopsy. Surgical approach included open and laparoscopy. Neurovascular bundle (NVB) was preserved in selective cases in consideration of patients’ clinical stage and request for post-operative sexual activity, and the indication of lymph node dissection included pre-operative risk stratification, MRI information and patients’ general condition. No neoadjuvant hormonal therapy was carried, and adjuvant hormonal therapy or radiotherapy were considered in proper indication. Post-operative phosphodiesterase type5 inhibitors (PDE5i) was selectively used based on patient’s request and clinician’s evaluation.

### Evaluation

All blood samples including those for measurements of sexual hormones were collected between 7 am and 9 am. TT and sex hormone-binding globulin (SHBG) were measured by chemiluminescence immunoassay (CLIA) using commercial available kit (Beckman Coulter, Fullerton, CA, USA). FT was calculated using Vermeulen’s formula [17]. The cut-off for low TT and low FT were set as lower than 300 ng/dl and 6.5 ng/dl according to relevant guideline [18].

Tumor stage and Gleason score were defined according to the 2010 American Joint Committee on Cancer TNM classification system. Positive surgical margin (PSM) was defined as the presence of cancer at the inked surface during the pathological evaluation of the final specimen. The blood sample for prostate-specific antigen (PSA) value were collected before biopsy. The presence of co-morbidities such as diabetes, hypertension and coronary heart disease were judged by relevant specialists.

Self-administered questionnaires were collected before and after surgery to evaluate continence and erectile function.

Continence and erectile function before and after were evaluated by self-administered questionnaires, in an attempt to decrease bias owing to patient–surgeon relationships [19]. Incontinence was defined as the requirement of more than 1 pad each day [20] Erectile dysfunction (ED) was defined as a lower than 17 points in the International Index of Erectile Function questionnaire 5 (IIEF-5) based on previous similar study [21], besides the cut-off of 12 points was also used since it’s a generally accepted criteria to differentiate mild or moderate ED [22].

### Follow-up regime, data collection and statistical analysis

Follow-up included PSA test monthly for the first 6 months, and every six months thereafter, and pelvic MRI or bone scan when indicated. The questionnaire or incontinence and ED were also collected simultaneously. Biochemical recurrence (BCR) was defined as any two consecutive increases in serum PSA over 0.2 ng/ml [23]. The definition of post-operative incontinence and ED were based on questionnaire results at 12 months after surgery.

Medians and quartiles for continuous measures and frequencies and percentages for categorical factors were used for the summary of patient characteristics. All statistical analysis was performed by SPSS 20.0 (IBM Corp, Armonk, NY, USA). Mann-Whitney U test and chi-square test were used for analysis of continuous s and dichotomous variables, respectively. Binary logistic regression was used to calculate risk factors for adverse pathological results and post-operative ED or incontinence, and the odds ratio as well as the 95% confidence interval would be shown. The receiver operating characteristic (ROC) curve was used to determine the optimum threshold. Kaplan-Meier curve using log-rank test was used to indicate the impact of TT and FT on BCR-free survival. Univariate and multivariate Cox regression models were performed for independent risk factors for BCR and the Wald (backward) model was used.

## Results

### Basic demographics and the distribution of TT and FT

**Fig. 1:**
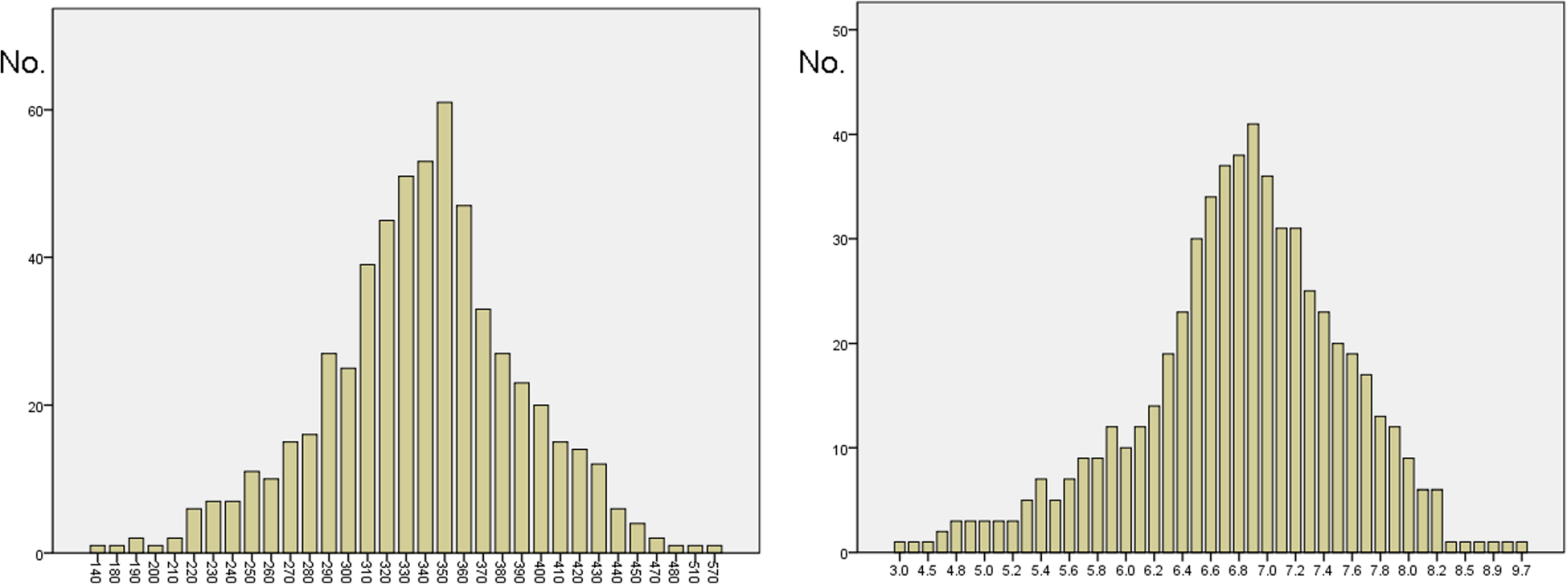
The distribution of pre-operative values of total testosterone (left, x-axis = total testosterone value, ng/dl) and free testosterone (right, x-axis = free testosterone value, ng/dl). Y-axis = number of patients

Altogether 586 patients were included and the median age was 69 years (interquartile IQR 63–73). The median TT was 344 ng/dL (IQR 314–374), with 106 patients (18.1%) lower than 300 ng/dl; the median FT was 6.9 ng/dL (IQR 6.4–7.3), with 152 patients (25.9%) lower than 6.5 ng/dl (Fig.1). 101 patients (17.2%) presented with both low TT and low FT.

The relationship of testosterone value with other clinical information was listed in Table 1. The presence of low TT and low FT were both related to older age (both *p* < 0.001), concomitant diabetes (*p* = 0.018 & 0.049), higher possibility of pre-operative ED (both *p* < 0.001), higher pre-operative PSA value (both *p* < 0.001), higher clinical stage (both *p* < 0.001) and higher Gleason score in biopsy (both *p* < 0.001). Besides patients with low TT tended to have the history of alcohol drinking (*p* < 0.001) and lower percentage of free-PSA (*p* = 0.022).

**Table 1:** Clinical and pathological characteristics of all patients stratified by total and free testosterone

	All	Total T			Free T		
		Low	Normal	*p* value	Low	Normal	*p* value
Patients, no.(%)	586(100)	106(18.1)	480(81.9)		152(25.9)	434(74.1)	
General information							
Age, no.(%)				< 0.001*			< 0.001*
< 70	297(50.7)	25(23.6)	272(56.7)		51(33.6)	246(56.7)	
≥ 70	289(49.3)	81(76.4)	208(43.3)		101(66.4)	188(43.3)	
Age, mean ± SD		71.73 ± 6.19	66.80 ± 8.07	< 0.001*	70.26 ± 7.18	66.79 ± 8.06	< 0.001*
BMI, mean ± SD		23.54 ± 3.63	23.40 ± 3.57	0.803	23.95 ± 3.50	23.46 ± 3.60	0.153
Diabetes, no.(%)				0.018*			0.049*
Absent	480(81.9)	78(73.6)	402(83.8)		116(76.3)	364(83.9)	
Present	106(18.1)	28(26.4)	78(16.2)		36(23.7)	70(16.1)	
Hypertension, no.(%)				0.764			0.895
Absent	498(85.0)	89(84.0)	409(85.2)		130(85.5)	368(84.8)	
Present	88(15.0)	17(16.0)	71(14.4)		22(14.5)	66(15.2)	
Coronary heart disease, no.(%)				0.316			0.460
Absent	519(88.6)	91(85.8)	428(89.2)		132(86.8)	387(89.2)	
Present	67(11.4)	15(14.2)	52(10.8)		20(13.2)	47(10.8)	
Smoking, no.(%)				0.301			0.426
Absent	495(84.5)	86(81.1)	409(85.2)		133(87.5)	366(84.3)	
Present	91(15.5)	20(18.9)	71(14.8)		19(12.5)	68(15.7)	
Alcohol consumption, no.(%)				0.001*			0.071
Absent	430(73.4)	63(59.4)	367(76.5)		103(67.8)	327(75.3)	
Present	156(26.6)	43(40.6)	113(23.5)		49(32.2)	107(24.7)	
Pre-operative ED, no.(%)				< 0.001*			< 0.001*
ED absent	376(64.2)	44(41.5)	332(69.2)		76(50.0)	300(69.1)	
ED present	210(35.8)	62(58.5)	148(30.8)		76(50.0)	134(30.9)	
Pre-operative tumor characteristics							
PSA, no.(%)				< 0.001*			< 0.001*
< 10 ng/ml	214(36.5)	11(10.4)	203(42.3)		35(23.0)	179(41.2)	
≥ 10 ng/ml	372(63.5)	95(89.6)	277(57.7)		117(77.0)	255(58.8)	
PSA, mean ± SD		16.34 ± 6.81	13.06 ± 6.90	< 0.001*	15.10 ± 6.92	13.14 ± 6.95	< 0.001*
Proportion of free-PSA, no.(%)				0.155			0.297
< 0.16	419(71.5)	82(77.4)	337(70.2)		114(75.0)	305(70.3)	
≥ 0.16	167(28.5)	24(22.6)	143(29.8)		38(25.0)	129(29.7)	
Proportion of free-PSA, mean ± SD		0.124 ± 0.060	0.139 ± 0.067	0.022*	0.132 ± 0.070	0.137 ± 0.064	0.144
Clinical tumor stage, no.(%)				< 0.001*			< 0.001*
T2a	79(13.5)	15(14.2)	64(13.3)		17(11.2)	62(14.3)	
T2b	154(26.3)	20(18.9)	134(27.9)		26(17.1)	128(29.5)	
T2c	219(37.4)	30(28.3)	189(39.4)		56(36.8)	163(37.6)	
T3	134(22.9)	41(38.7)	93(19.4)		53(34.9)	81(18.7)	
Biopsy Gleason Score, no.(%)				< 0.001*			< 0.001*
6	143(24.4)	10(9.4)	133(27.7)		16(10.5)	127(29.3)	
7	295(50.3)	52(49.1)	243(50.6)		76(50.0)	219(50.5)	
8 or higher	148(25.3)	44(41.5)	104(21.7)		60(39.5)	88(20.3)	
Post-operative information							
Surgical approach, no.(%)				0.067			0.257
Open	272(46.4)	58(54.7)	214(44.6)		77(50.7)	195(44.9)	
Laparoscopic	314(53.6)	48(45.3)	266(55.4)		75(49.3)	239(55.1)	
NVB preserve, no.(%)				0.913			0.771
Not preserved	224(38.2)	41(38.7)	183(38.1)		60(39.5)	164(37.8)	
Preserved	362(61.8)	65(61.3)	297(61.9)		92(60.5)	270(62.2)	
Pathological tumor stage, no.(%)				0.006*			< 0.001*
T2a	76(13.0)	13(12.3)	63(13.1)		13(8.6)	63(14.5)	
T2b	150(25.6)	18(17.0)	132(27.5)		28(18.4)	122(28.1)	
T2c	180(30.7)	28(26.4)	152(31.7)		41(27.0)	139(32.0)	
T3	180(30.7)	47(44.3)	133(27.7)		70(46.1)	110(25.3)	
Final Gleason Score, no.(%)				< 0.001*			< 0.001*
6	126(21.5)	10(9.4)	116(24.2)		11(7.2)	115(26.5)	
7	259(44.2)	23(21.7)	236(49.2)		47(30.9)	212(48.8)	
8 or higher	201(34.3)	73(68.9)	128(26.7)		94(61.8)	107(24.7)	
Seminal invasion, no.(%)				0.022*			0.026*
Absent	563(96.1)	97(91.5)	466(97.1)		141(92.8)	422(97.2)	
Present	23(3.9)	9(8.5)	14(2.9)		11(7.2)	12(2.8)	
PSM, no.(%)				0.128			0.094
Absent	536(91.5)	93(87.7)	443(92.3)		134(88.2)	402(92.6)	
Present	50(8.5)	13(12.3)	37(7.7)		18(11.8)	34(7.8)	
Lymph node status, no.(%)				0.003*			0.001*
N-	225(38.4)	52(49.1)	173(36.0)		68(44.7)	157(36.2)	
Nx	336(57.3)	46(43.4)	290(60.4)		71(46.7)	265(61.1)	
N+	25(4.3)	8(7.5)	17(3.6)		13(8.6)	12(2.8)	
Post-operative ED, no.(%)				0.001*			< 0.001*
ED absent	197(33.6)	13(12.3)	184(38.3)		22(14.5)	175(40.3)	
ED present	179(30.5)	31(29.2)	148(30.8)		54(35.5)	125(28.8)	
Post-operative incontinence, no.(%)				0.506			0.662
Absent	511(87.2)	94(88.7)	417(86.9)		134(88.2)	377(86.9)	
Present	69(11.8)	10(9.4)	59(12.3)		16(10.5)	53(12.2)	

*PSA* prostate-specific antigen; *BMI* body mass index; *ED* erectile dysfunction; *NVB* neurovascular bundle; *PSM* positive surgical margin

*p* value of less than 0.05 are in boldface

*Statistically significant

### Pathological outcomes

By pathological examination after radical prostatectomy, 180 patients (30.7%) were found to suffer from T3 stage disease, including 23 patients (3.9%) with seminal invasion. A Gleason score of 8 or higher was noticed in 201 patients (34.3%). Positive surgical margin was present in 50 patients (8.5%). Lymph node dissection was performed in 250 patients (42.7%) and positive lymph node was found in 25 patients (4.3%).

The binary logistic analysis for independent risk factors for adverse pathological outcomes was shown in Table 2. Patients with low FT had higher risk for pT3 (*p* = 0.020) and high Gleason score (*p* = 0.011), and patients with low TT also had higher chance for high Gleason score (*p* = 0.045). PSA value, clinical tumor stage and Gleason score in biopsy were also related to those worse pathological findings. Rerunning the dataset by including only FT or only TT in logistic regression for high Gleason score produced the same results.

**Table 2:** Logistic regression for risk factors for adverse pathological outcomes

Variables	pT3			Gleason 8 or higher		
	OR	95%CI	*p* value	OR	95%CI	*p* value
TT (normal vs low)	1.017	0.479–2.159	0.965	0.484	0.237–0.985	0.045*
FT (normal vs low)	0.587	0.374–0.921	0.020*	0.456	0.249-0.835	0.011*
Age (continuous)	1.006	0.979–1.034	0.665	1.006	0.979–1.003	0.677
BMI (continuous)	1.016	0.960–1.076	0.581	1.014	0.960–1.072	0.607
Diabetes (presence vs absence)	1.243	0.719–2.149	0.436	0.970	0.573–1.641	0.909
Hypertension (presence vs absence)	0.855	0.472–1.549	0.605	1.087	0.629–1.878	0.765
Coronary heart disease (presence vs absence)	1.203	0.624–2.322	0.581	1.279	0.683–2.395	0.443
Smoke (presence vs absence)	1.280	0.723–2.266	0.397	1.400	0.825–2.376	0.212
Alcohol (presence vs absence)	0.763	0.471–1.236	0.272	1.428	0.924–2.208	0.109
PSA (≥10 ng/ml vs < 10 ng/ml)	1.007	0.644–1.575	0.975	1.829	1.195–2.800	0.005*
Proportion of free-PSA (≥ 0.16 vs < 0.16)	0.851	0.540–1.340	0.486	1.030	0.664–1.599	0.894
Clinical Tumor stage (T2a vs T3)	0.057	0.021–0.153	< 0.001*	0.439	0.212-0.909	0.027*
Clinical (T2b vs T3)	0.059	0.029–0.120	< 0.001*	0.454	0.258-0.800	0.006*
Clinical (T2c vs T3)	0.474	0.299–0.750	0.001*	0.840	0.513-1.374	0.487
Biopsy Gleason Score (6 vs 8 or higher)	0.289	0.149–0.562	< 0.001*	0.179	0.093-0.344	< 0.001*
Biopsy Gleason Score (7 vs 8 or higher)	0.800	0.500–1.279	0.351	0.723	0.467–1.121	0.148

*OR* odds ratio; *CI* confidence interval; *TT* total testosterone; *FT* free testosterone; *PSA* prostate-specific antigen; *BMI* body mass index; *PSM* positive surgical margin

*p* value of less than 0.05 are in boldface

*Statistically significant

### Survival results

**Fig. 2:**
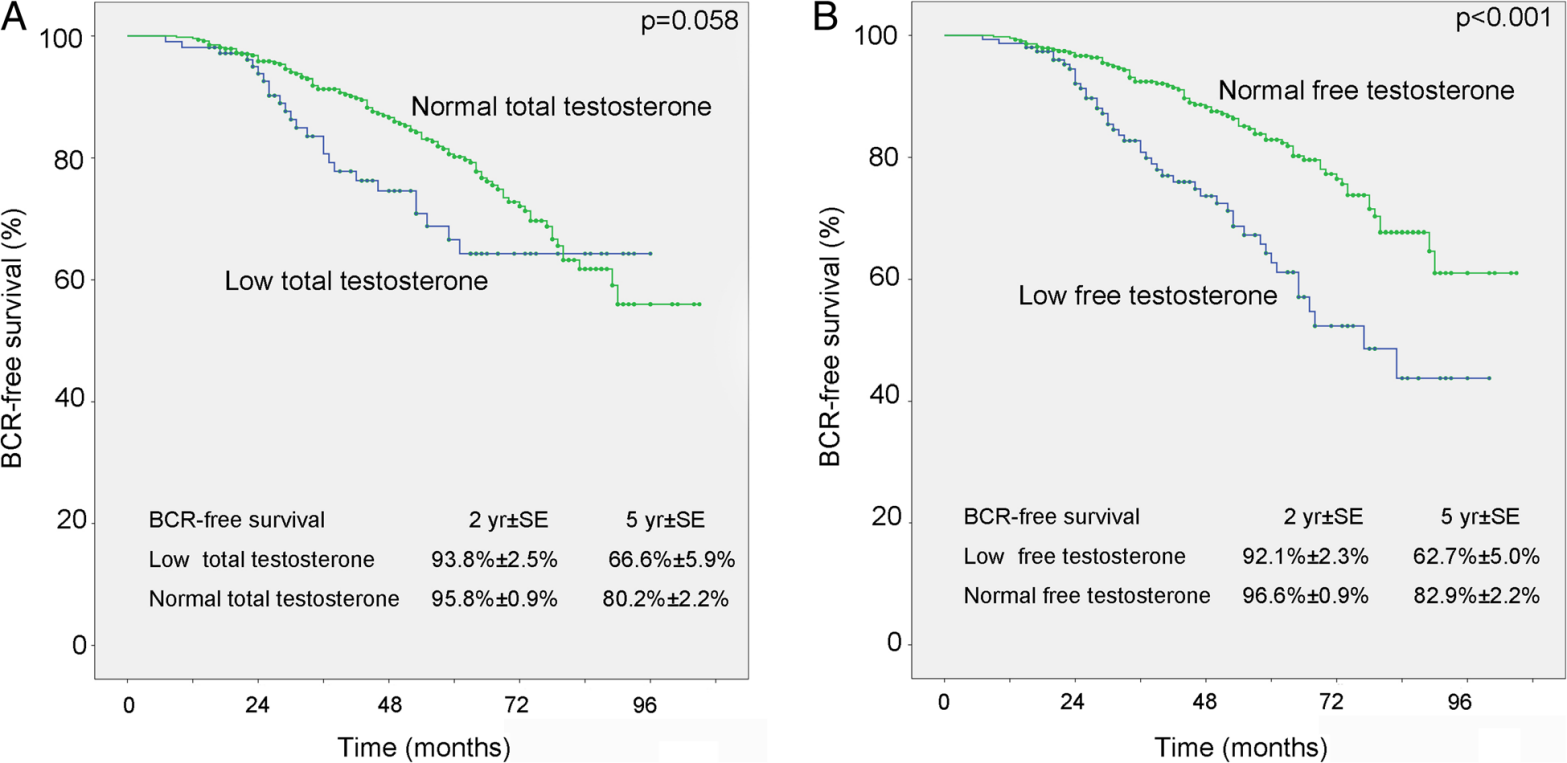
Estimated Kaplan-Meier curves in predicting biochemical recurrence-free survival stratified by total testosterone (**a**, *p* = 0.058) or free testosterone (**b**, *p* < 0.001). BCR = biochemical recurrence

The median follow-up duration was 52 moths (IQR 29–67). BCR was noticed in 119 patients (20.3%). 39 patients (6.7%) died, including 31 died from late-stage prostate cancer. Kaplan-meire curves indicated a higher risk for BCR in patients with lower FT (*p* < 0.001), while although there’s a trend towards higher proportion of BCR in patients with lower TT, no statistical significance was found (*p* = 0.058) (Fig. 2). The relationship between FT for BCR was confirmed by univariate and multivariate Cox regression analysis (*p* < 0.001 & *p* = 0.005), together with pathological stage and Gleason score. (Table 3).

**Table 3:** Risk factors for biochemical recurrence-free survival

Variables	Univariate analysis			Multivariate analysis		
	HR	95%CI	p value	HR	95%CI	*p* value
Total T (normal vs low)	0.655	0.421–1.019	0.061			
Free T (normal vs low)	0.434	0.299–0.628	< 0.001*	0.564	0.377-0.843	0.005*
Age (continuous)	1.019	0.995–1.045	0.126			
BMI (continuous)	0.990	0.941–1.042	0.710			
Diabetes (presence vs absence)	0.888	0.549–1.436	0.628			
Hypertension (presence vs absence)	0.788	0.442–1.403	0.417			
Coronary heart disease (presence vs absence)	0.878	0.483–1.596	0.670			
Smoke (presence vs absence)	0.657	0.382–1.130	0.129			
Alcohol (presence vs absence)	0.982	0.646–1.493	0.931			
PSA (≥10 ng/ml vs < 10 ng/ml)	1.515	1.020–2.250	0.039*	1.261	0.835-1.904	0.270
Proportion of free-PSA (≥ 0.16 vs < 0.16)	0.794	0.527–1.196	0.296			
Surgical approach (laparoscopic vs open)	0.922	0.643–1.322	0.658			
NVB preserve(presence vs absence)	0.935	0.648–1.349	0.719			
Pathological Tumor stage (T2a vs T3)	0.270	0.116–0.626	0.002*	0.333	0.128-0.804	0.014*
Pathological Tumor stage (T2b vs T3)	0.414	0.252–0.677	< 0.001*	0.457	0.268-0.780	0.004*
Pathological Tumor stage (T2c vs T3)	0.633	0.415–0.966	0.034*	0.814	0.523-1.268	0.363
Final Gleason Score (6 vs 8 or higher)	0.316	0.173–0.577	< 0.001*	0.532	0.278-1.019	0.057
Final Gleason Score (7 vs 8 or higher)	0.518	0.353–0.761	0.001*	0.647	0.431-0.971	0.036*
Lymph node status (N- vs N+)	0.328	0.167–0.644	0.001*	0.523	0.259-1.054	0.070
Lymph node status (Nx vs N+)	0.407	0.215–0.768	0.006*	0.927	0.467-1.840	0.829
Seminal invasion (presence vs absence)	2.483	1.255–4.914	0.009*	1.048	0.497-2.211	0.902
PSM (presence vs absence)	2.350	1.453–3.803	0.001*	1.432	0.862-2.378	0.165

*HR* Hazard Ratio; *CI* confidence interval; *TT* total testosterone; *FT* free testosterone; *PSA* prostate-specific antigen; *BMI* body mass index; *PSM* positive surgical margin; *NVB* neurovascular bundle

*p* value of less than 0.05 are in boldface

*Statistically significant

### Continence and erectile function in follow-up

By pre-operative evaluation, 10 patients (1.7%) were considered as possible incontinence; 210 patients (35.8%) were defined as ED including a number of patients declared no requirement of sexual life. They were excluded in the analysis of risk factors for post-operative incontinence or post-operative impotence.

In 12 months after surgery, 69 patients (11.8%) required one or more pads everyday due to incontinence. The value of TT and FT was not related to the occurrence of incontinence (*p* = 0.409 & 0.784, Table 4). Only older age was associated with higher risk of incontinence (*p* = 0.034).

**Table 4:** Logistic regression for risk factors for incontinence and impotence

Variables	Pre-operative ED			Post-operative ED			Post-operative incontinence		
	OR	95%CI	*p* value	OR	95%CI	*p* value	OR	95%CI	*p* value
TT (normal vs low)	0.525	0.322–0.856	0.010*	1.580	0.491-5.076	0.443	1.566	0.540–4.540	0.409
FT (normal vs low)	0.824	0.433–1.570	0.557	0.332	0.180–0.612	< 0.001*	0.885	0.368–2.128	0.784
Age(continuous)	1.107	1.074–1.141	< 0.001*	1.118	1.076-1.161	< 0.001*	1.039	1.003-1.076	0.034*
BMI (continuous)	0.968	0.918–1.021	0.232	0.973	0.911–1.039	0.493	1.033	0.961–1.111	0.377
Diabetes (presence vs absence)	0.846	0.519–1.378	0.502	1.073	0.573–2.008	0.826	1.095	0.543–2.210	0.800
Hypertension (presence vs absence)	0.784	0.457–1.347	0.379	0.760	0.404–1.428	0.393	1.115	0.549–2.265	0.764
Coronary heart disease (presence vs absence)	1.443	0.803–2.594	0.221	0.732	0.336–1.596	0.433	0.813	0.346–1.909	0.634
Smoke (presence vs absence)	2.450	1.477–4.064	0.001*	0.720	0.352-1.470	0.366	0.794	0.370–1.705	0.555
Alcohol (presence vs absence)	1.197	0.768–1.867	0.427	1.102	0.617–1.969	0.742	0.800	0.421–1.522	0.496
PSA (≥10 ng/ml vs < 10 ng/ml)	1.511	0.997–2.289	0.052	0.806	0.495–1.311	0.384	0.842	0.486–1.459	0.539
Proportion of free-PSA (≥ 0.16 vs < 0.16)	1.333	0.878–2.023	0.177	0.899	0.530–1.527	0.694	0.931	0.523–1.656	0.807
Clinical Tumor stage (T2a vs T3)	0.479	0.244–0.939	0.032*						
Clinical (T2b vs T3)	0.949	0.558–1.613	0.847						
Clinical (T2c vs T3)	0.655	0.400–1.072	0.092						
Biopsy Gleason Score (6 vs 8 or higher)	0.829	0.470–1.462	0.518						
Biopsy Gleason Score (7 vs 8 or higher)	0.823	0.523–1.294	0.398						
Surgical approach (open vs laparoscopic)				0.616	0.385–0.986	0.044*	0.972	0.580-1.628	0.913
NVB preserve(presence vs absence)				1.479	0.890–2.456	0.131	1.069	0.621–1.840	0.809
Pathological Tumor stage (T2a vs T3)				0.806	0.350–1.859	0.614	1.614	0.634–4.112	0.316
Pathological Tumor stage (T2b vs T3)				1.096	0.553–2.171	0.794	1.700	0.793–3.645	0.173
Pathological Tumor stage (T2c vs T3)				1.177	0.638–2.196	0.602	1.274	0.617–2.629	0.513
Final Gleason Score (6 vs 8 or higher)				0.555	0.280–1.100	0.092	1.134	0.513–2.510	0.756
Final Gleason Score (7 vs 8 or higher)				0.466	0.263–0.826	0.009*	0.976	0.514–1.852	0.940
Lymph node status (N- vs N+)				2.242	0.638–7.877	0.208	3.202	0.393–26.079	0.277
Lymph node status (Nx vs N+)				3.344	0.962–11.623	0.058	2.864	0.349–23.489	0.327
PSM (presence vs absence)				0.722	0.304–1.716	0.460	1.005	0.364–2.773	0.993

*OR* odds Ratio; *CI* confidence interval; *ED* erectile dysfuncion;*TT* total testosterone; *FT* free testosterone; *PSA* prostate-specific antigen; *BMI* body mass index; *PSM* positive surgical margin; *NVB* neurovascular bundle

*p* value of less than 0.05 are in boldface

*Statistically significant

Patients with satisfactory erectile function dropped from 376 (64.2%) before surgery to 197 (33.6%) in 12 months after surgery. Patients with low TT value were more likely to exhibit pre-operative ED (*p* = 0.010), together with older age (*p* < 0.001) and smoking history (*p* = 0.001), while low FT was related to post-operative ED (p < 0.001) together with older age (*p* < 0.001) (Table 4). There are also statistically significant relationships between tumor stage, surgical approach, Gleason score, and ED.

**Fig. 3:**
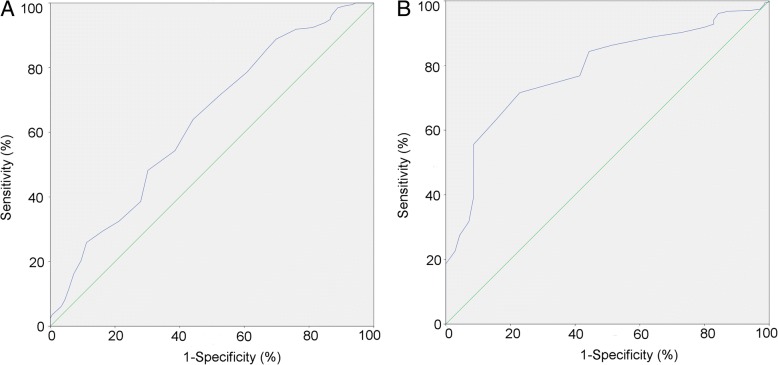
ROC curves in predicting erectile function by free testosterone value (**a** predicting IIEF-5 over 17 points, AUC = 0.634; **b** predicting IIEF-5 over 12 points, AUC = 0.782). ROC = receiver operating characteristic; AUC = area under curve Note: since higher free testosterone value was related to better erectile function, the ROC curves are showing the prediction of having the ability for sexual life, NOT the occurrence of ED.

The ROC curve indicating the relationship between pre-operative FT value and post-operative erectile function was shown in Fig. 3A, with an AUC of 0.634. It’s notable that a cut-off of 6.85 ng/dL would lead to the highest Youden index of 1.198. We also considered defining ED as lower than 12 points in IIEF-5, which lead to a significant better predictive value of FT with an AUC of 0.782 (Fig. 3B).

## Discussion

The results of the current study demonstrated that FT was a useful pre-operative marker for oncologic outcomes and quality of life. It’s more sensitive than TT since patients with low FT might have higher risk for BCR and post-operative ED. Therefore, FT could be used in clinical practice as a pre-operative marker for risk-stratification in future clinical practice. Patients with low FT should be informed of the possible high risk for disease relapse, and less possibility of erectile function recovery. More aggressive treatment measures could be considered by the treating physician, including lymph node dissection, closer surveillance and timely adjuvant therapy, and a prophylactic PDE5i could be an important measure for those desired for post-operative sexual activity.

In this study low TT value was also related to some adverse clinical and pathological features, and despite no statistical significance, patients with low TT value tended to suffer from high risk of BCR and post-operative ED. These results were in accordance with the majority of the previous publications, and a number of mechanisms have been raised to explain the link between low TT value and tumor aggressiveness [7–9]. But still it’s notable that different results existed regarding this issue [4], and Albuquerque et al. attribute this controversy to demographic variability between cohorts, and the possible co-existence of other undefined mechanisms [24]. Based on the results of the current study, we hypothesize that perhaps it’s FT that regulates the development and progression of PCa, and different proportion of FT in patients in previous studies might be the explanation for various results.

The majority of TT was binding to SHBG, resulting in an inactivated status, thus FT is actually responsible for the biological activity [12, 16]. Therefore, the low FT value would indicate an active androgen-depleted environment, which could function as serious promoter factors for developing more aggressive PCa. On the other hand, low FT value would certainly reflecting the possibility of accompanying MetS, which would regulate the aggressiveness of PCa [3, 25]. Thus high Gleason score and correspondingly, high tumor stage and higher risk of BCR were noted in patients with low FT levels. The underlying detailed mechanisms required more investigation, and we suggest future studies regarding late onset hypogonadism (LOH) and PCa should pay special attention to FT.

TT was commonly regarded to be important to exhibit the presence of LOH and thus erectile function in aged patients without PCa, but the relationship with continence or erectile function recovery in patients underwent RP was never investigated, not to mention FT. The current results showed that low FT value was related to post-operative impotence. Clinicians could warn the high-risk for erectile dysfunction after surgery, and plan recovery treatment such as tadalafil. Besides the results demonstrated the importance of FT value in the evaluation of aged man with LOH or ED, even in patients without PCa and it’s interesting that recent literatures have demonstrated the association between ED and subsequent PCa development [26]; more clinical and basic investigations would be required to fully illustrate the relationship.

Our study is the first study that illustrated the relationship between FT and the prognosis after RP, and the first investigation about the impact of androgen on post-operative quality of life. Yet it still has some limitations. The main limitation of this study was related to its retrospective nature, thus some important factors were unavailable, including, waist circumference, urodynamic examinations and post-operative hormonal tests. Some advanced techniques and managements were not carried, including neoadjuvant hormonal treatment or robotic surgery; besides the indications for lymph node dissection, the preservation of NVB and the use of PDE5i were not standardized. Further external validation are required to address these limitations.

## Conclusions

Pre-operative low FT was related to adverse pathological outcomes, biochemical recurrence and post-operative ED for patients underwent radical prostatectomy. This test could help risk-stratification and plan individualized treatment. More basic researches would be required to illustrate the mechanisms and further validation would be necessary.

## Abbreviations

ADH: Androgen-deprivation treatment
BMI: body mass index
FT: Free testosterone
IIEF-5: International Index of Erectile Function questionnaire 5
LOH: late onset hypogonadism
MetS: metabolic syndrome
NVB: Neurovascular bundle
PCa: prostate cancer
PDE5i: phosphodiesterase type5 inhibitors
PSA: prostate-specific antigen
PSM: Positive surgical margin
RP: radical prostatectomy
TT: total testosterone
